# A 3D-Printed Self-Learning Three-Linked-Sphere Robot for Autonomous Confined-Space Navigation

**DOI:** 10.1002/aisy.202100039

**Published:** 2021-06-26

**Authors:** Brian Elder, Zonghao Zou, Samannoy Ghosh, Oliver Silverberg, Taylor E. Greenwood, Ebru Demir, Vivian Song-En Su, On Shun Pak, Yong Lin Kong

**Affiliations:** Department of Mechanical Engineering, University of Utah, Salt Lake City, UT 84112, USA; Department of Mechanical Engineering, Santa Clara University, Santa Clara, CA 95053, USA; Department of Mechanical Engineering, University of Utah, Salt Lake City, UT 84112, USA; Department of Mechanical Engineering, Santa Clara University, Santa Clara, CA 95053, USA; Department of Mechanical Engineering, University of Utah, Salt Lake City, UT 84112, USA; Department of Mechanical Engineering, Santa Clara University, Santa Clara, CA 95053, USA; Department of Mechanical Engineering, University of Utah, Salt Lake City, UT 84112, USA; Department of Mechanical Engineering, Santa Clara University, Santa Clara, CA 95053, USA; Department of Mechanical Engineering, University of Utah, Salt Lake City, UT 84112, USA

**Keywords:** 3D printing, confined-space navigation, reinforcement learning, robots, three-linked-sphere

## Abstract

Reinforcement learning control methods can impart robots with the ability to discover effective behavior, reducing their modeling and sensing requirements, and enabling their ability to adapt to environmental changes. However, it remains challenging for a robot to achieve navigation in confined and dynamic environments, which are characteristic of a broad range of biomedical applications, such as endoscopy with ingestible electronics. Herein, a compact, 3D-printed three-linked-sphere robot synergistically integrated with a reinforcement learning algorithm that can perform adaptable, autonomous crawling in a confined channel is demonstrated. The scalable robot consists of three equally sized spheres that are linearly coupled, in which the extension and contraction in specific sequences dictate its navigation. The ability to achieve bidirectional locomotion across frictional surfaces in open and confined spaces without prior knowledge of the environment is also demonstrated. The synergistic integration of a highly scalable robotic apparatus and the model-free reinforcement learning control strategy can enable autonomous navigation in a broad range of dynamic and confined environments. This capability can enable sensing, imaging, and surgical processes in previously inaccessible confined environments in the human body.

## 1. Introduction

Electronics and robots can address a broad range of unmet clinical needs, from probing previously inaccessible regions of the human body to assisting in complex surgical processes.^[1-3]^ Many biomedical robots have been developed for use at the microscale in various scenarios, including for noninvasive surgery^[4-6]^ and targeted therapies.^[7-9]^ Mobile, microscale robots (“microbots”) are typically designed to perform a specific gait in a specific environment.^[7]^ However, the physical properties of biological fluids, tissues, and structures vary significantly,^[10-12]^ and biological environments are often both dynamic and uncertain. For example, a gait designed for an ingestible robot to perform in one environment may not be effective in another. In addition to challenges in localization,^[13]^ the complexity and uncertainty of biological environments are also challenging to model. Robots controlled via conventional control methods (e.g., proportional–integral–derivative control) often are incapable of effective locomotion in these dynamic environments.

As an alternative control method, reinforcement learning, can impart robots with the ability to discover effective behavior based on their interactions with the unknown environment. Reinforcement learning is thus an advantageous control method in biomedical applications because it can reduce modeling and sensing requirements and enable robots to adapt to environmental changes. Reinforcement learning has been used for gait optimization in crawling robots to discover movement policies for traversing various substrates or confined spaces using complex legged and wheeled robots.^[14-20]^ Previous works of reinforcement-learning microbots have included hexapod microbots with adaptable gait policies,^[14,15]^ controllable swarms of optical tweezer robots,^[21]^ microscale biomedical robots,^[7,22-24]^ and adaptive industrial inspection robots.^[18-20]^

In addition to being complex, dynamic, and uncertain, most biological environments are also confined. For example, robot-assisted endoscopy in the gastrointestinal system requires a robot not only to be able to perform functional tasks but also to fit and navigate effectively in a confined space. However, the currently demonstrated reinforcement-learning robots are based on complex designs that are inherently challenging to scale for effective navigation in confined space.^[25,26]^ Recent advances in 3D printing can increase the functional capacity and density of medical electronics and robots^[27-29]^ to enable navigation in a confined space.

**Figure 1.**
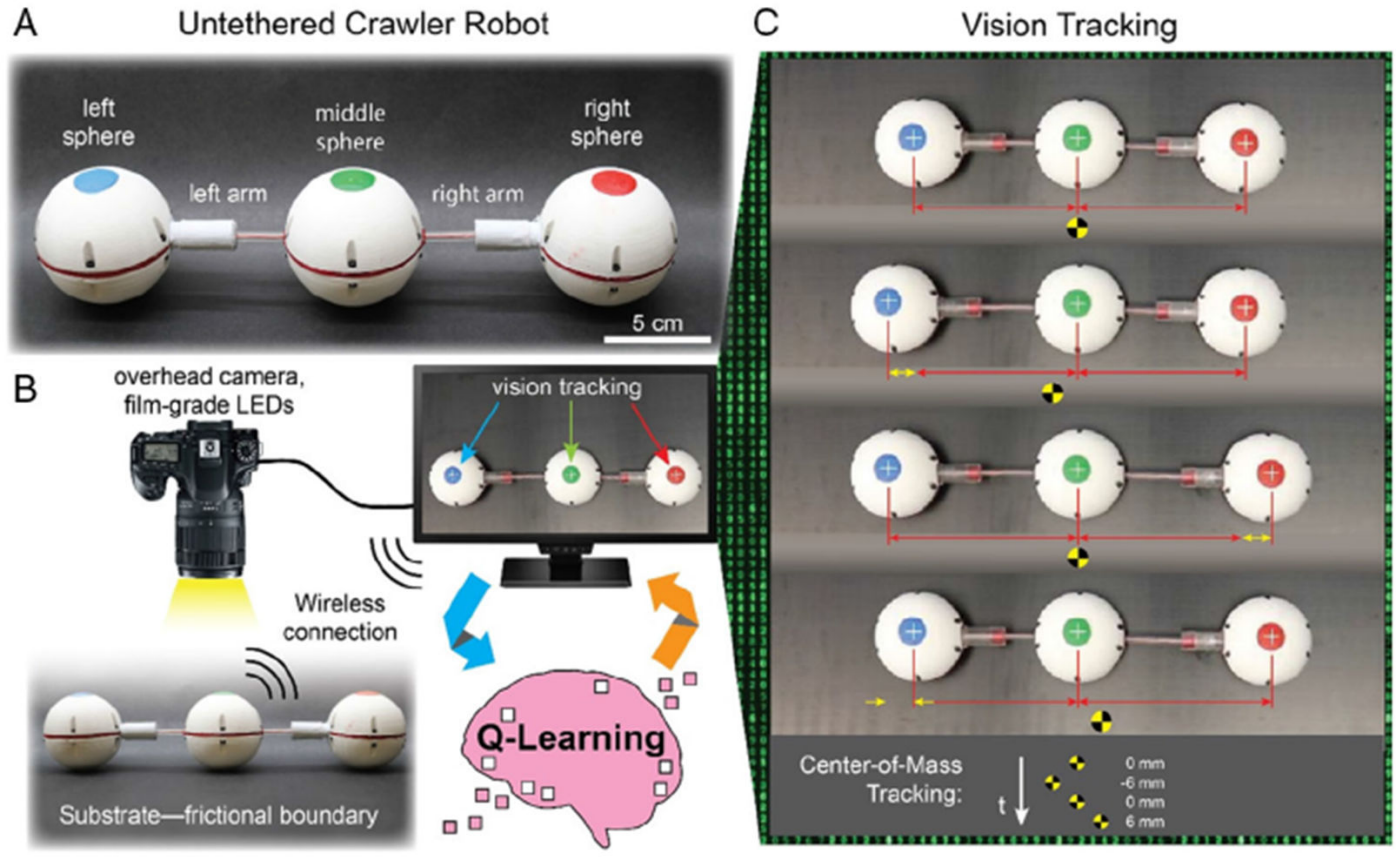
Untethered crawler robot integrated with a reinforcement-learning algorithm. A) The architecture of an untethered, three-linked-sphere robot that is internally powered with extendable linear actuators. B) Experimental setup for closed-loop reinforcement-learning control where the position tracking is achieved with an overhead camera and connected with a computer wirelessly over Bluetooth. C) Colored circles on the spheres are used to track positions (white cross overlays, placed by the visual tracking algorithm) and the center of mass (yellow and black Secchi disks).

In this work, we integrate an untethered and compact 3D-printed three-linked-sphere robot with a model-free reinforcement-learning algorithm (Figure 1). Specifically, our work is the first experimental integration of the theoretical Najafi–Golestanian three-linked-sphere mechanism with a reinforcement-learning algorithm as a highly scalable and relatively simple self-learning robot that can navigate in confined spaces. The algorithm used in this study is based on a standard Q-learning algorithm,^[30,31]^ which is a reinforcement-learning algorithm that can enable self-learning behavior without a model.^[32-34]^

First described by Najafi and Golestanian, the three-linked-sphere robot is one of the most basic architectures capable of performing nonreciprocal swimming gaits, which is essential for overcoming the challenge of swimming at low Reynolds numbers.^[35]^ However, gaits that are optimal for locomotion in one medium may become largely ineffective in a different medium. Therefore, the ability to adapt the gaits based on environmental changes is crucial for locomotion in complex, varying environments.^[36]^ The Najafi–Golestanian gait’s effectiveness for crawling on a frictional surface is first probed by programming the robot to execute this known policy. Reinforcement learning is then exploited to enable the robot to adapt its gaits based on its interactions with the frictional surface.

Utilizing a three-linked-sphere robot is desirable for biomedical applications because its form and motion are compact and inherently scalable. The robot’s body consists of three equally sized spheres linearly coupled by two prismatic joints. Extending or contracting the joints in specific sequences allows the robot to achieve locomotion. In contrast to prior works—which require complex mechanisms such as 12-degree-of-freedom (DOF) quadrupeds,^[17]^ 12-DOF^[15]^ and 18-DOF^[14,37]^ hexapods, multiple DOF segmented rectilinear^[16]^ or wheeled^[18]^ robots for locomotion that are challenging to miniaturize for use in a confined space—our work focuses on a highly simplified and scalable mechanism that enables locomotion in a confined space.

In principle, the robot can be readily rescaled while preserving its architecture. Within the millimeter regime of dry friction crawling, the robot’s size is not expected to affect the crawling motion. In contrast to prior work that requires complex gait mechanisms, the locomotion of the three-linked-sphere robot is enabled by an imbalance of resistive dry surface forces (Coulomb friction, electrostatic, Van der Waals) on either side of the extending or contracting links. In this work, as our goal is a proof-of-concept demonstration, we have chosen a length scale that allows us to leverage commercial off-the-shelf (COTS) electronics components. Our results obtained from this work are scalable to the future target length scale. The integration of smaller electronics is feasible but is beyond the scope of the current work.

**Figure 2.**
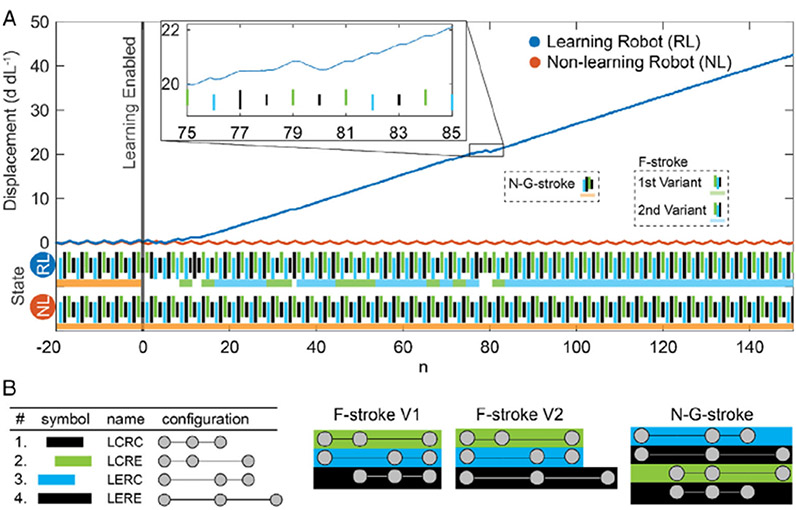
Gait adaption of the three-linked-sphere robots via Q-learning. A) The robots were first initialized with N—G strokes (n = −20 to 0). The reinforcement learning was turned on for the learning robot (RL) at n = 0, which was compared with a nonlearning robot (NL). The learning robot (blue) began to move using an F-stroke policy at n = 17 steps and achieved a mean velocity of 0.25 *d* d*L*^−1^ *n*^−1^. The learning robot fully learned the F-stroke policy in n = 54 steps. In contrast, the nonlearning robot (red) remained in the N—G stroke, and therefore its center of mass has no net displacement. The robot is considered to have learned a movement policy when the corresponding set of Q values remain higher than all other sets for n ≥ 48 steps. Policy adaptation is marked at the beginning of this period. The inset figure contains an enlarged view of a section of the learning robot’s displacement from n = 75 to 85. The color bars below the chart show the gait of the robot as described in part (B). Colored underlines indicate gait patterns, namely, the N—G stroke (orange) and the F-stroke (first variant green and second variant blue). B) Schematics of the robot states and archetypal strokes, color coded to match the state sequences in part (A).

The 3D-printed robot’s motion is controlled using a model-free reinforcement-learning control strategy called Q-learning. Using Q-learning, the robot identifies favorable gait policies by alternating between states, monitoring the corresponding displacement changes using remote visual sensing, and rewarding favorable motions based on a user-determined reward function. Q-learning is versatile and can be applied to any robot whose movement can be described by discrete states. The two joints’ discrete extension and contraction yield 4 geometry configurations (states), shown in Figure 2, and 12 state-action pairs. The advantage of the design is that the robot can be easily expanded to the *n*-linked-sphere crawler^[36,38]^ and the Parking three-linked-sphere crawler robots^[39,40]^ to achieve more complex movements. One of the key limitations in our approach is that with increasing complexity, the length of the adaptation period is exponential. Nevertheless, in future work, the adaptation period could possibly be reduced by first training the swimmer in a simulation and then using the trained result in a real environment.

In this study, we focus on a three-linked-sphere robot because of its simplicity, which can enable subsequent miniaturization in future designs. Its simplicity, as well as its scalability, is attractive for future biomedical applications. Experiments are performed to demonstrate that, using reinforcement learning, the three-linked-sphere robot is capable of adaptable, autonomous crawling in both open space and a confined channel.

In principle, our current proof-of-concept demonstration is also applicable to a robot created using alternative fabrication approaches (e.g., molding and subtractive manufacturing). In this example, 3D printing was used to fabricate several robot parts because: 1) most of the freeform components (e.g., spherical chassis, arm-coupling mechanism) would be challenging to fabricate via other approaches such as molding. 2) 3D printing allows us to control mechanical properties, such as surface friction (e.g., by tailoring the microfeatures on the surface), which is the fundamental driving mechanism of the three-linked-sphere robot. In future work, the freeform fabrication approach with 3D printing can enable a highly scalable, freeform integration with the advances of 3D-printed electronics^[27-29,41,42]^ or achieve a hybrid electronic integration approach by leveraging pick-and-place.^[43]^ Overall, in comparison to conventional processes such as molding, the freeform fabrication capability of 3D printing can enable a scalable fabrication methodology (within the limitation of the fabrication tool) and higher functional integration, which can better leverage the scalability of the three-linked sphere robot.

## 2. Experimental Results

### 2.1. Gait Adaptation

Gait adaptation was analyzed by comparing the difference in displacement, state sequence (strokes), and *Q* values between two sets of experiments: a *learning robot* controlled via reinforcement learning and a *nonlearning robot* that continuously performed a preset gait (stroke sequence). The nonlearning robot was programmed to perform the Najafi–Golestanian stroke^[35]^ (N—G stroke) that has previously been shown to enable locomotion in low-Reynolds-number fluids. The robots were placed on a horizontal frictional surface and traveled in one dimension (left to right). To simulate a transition in the environment and allow the Q-learning algorithm (Equation (1)) to make decisions, the learning robot was directed to begin using the N—G stroke and was influenced to continue this stroke through artificial positive rewards for the first 20 steps (*n* = −20 to 0). At step n = 0, the Q-learning algorithm was allowed to make decisions in the learning robot. Thus, after step n = 0, the learning robot explored other state actions and adapted its movement policy in ≈54 steps to a productive stroke sequence and achieved a mean normalized velocity *V*_m_ = 0.25 *d* d*L*^−1^ *n*^−1^. However, in the frictional surface environment, the N—G stroke was incapable of forward locomotion; the nonlearning robot remained at the starting location for the duration of the experiment (see Figure 2 and Movie S1, Supplementary Information).

### 2.2. Goal Adaptation

**Figure 3.**
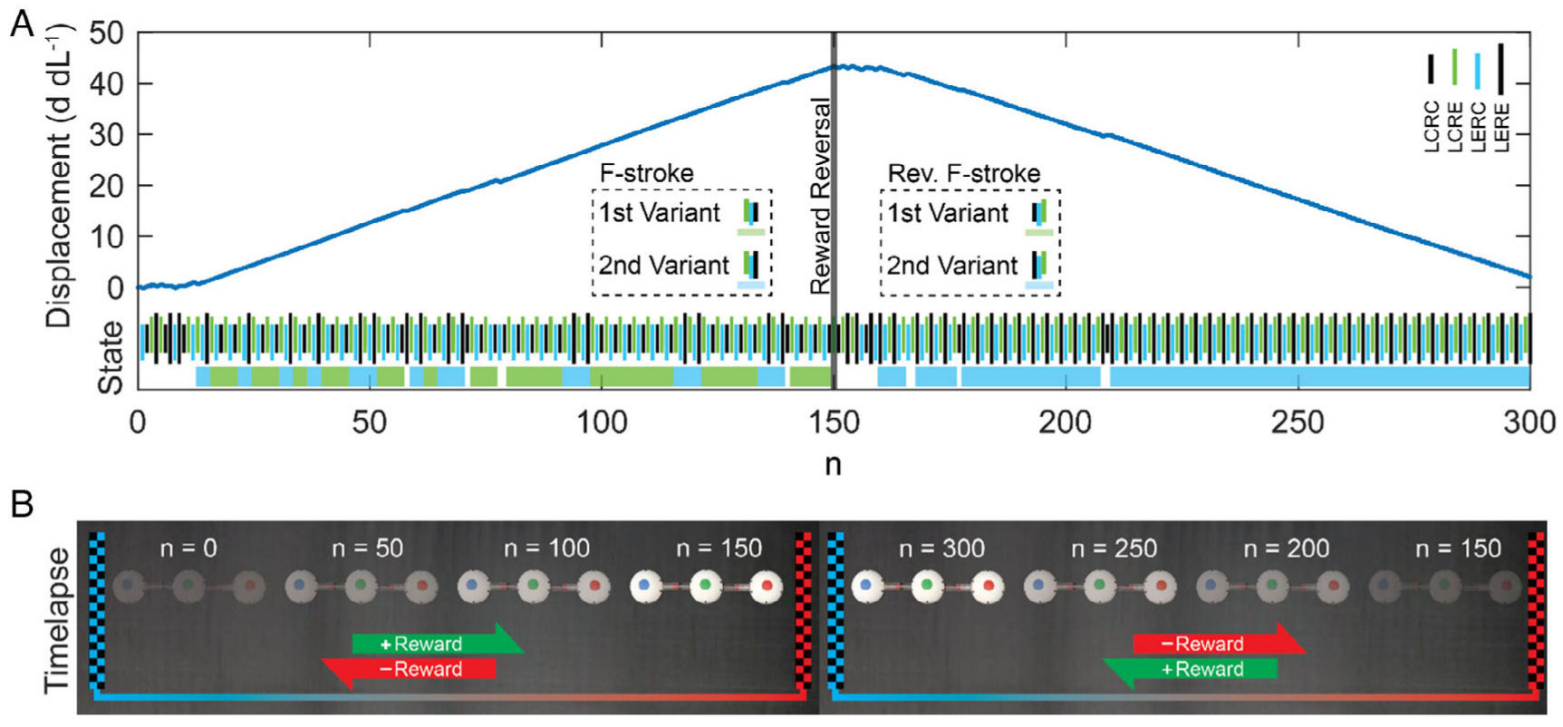
Reinforcement learning and adaptation during goal adjustment of the three-linked-sphere robots. A) The robot can unlearn a reinforced pattern (F-stroke) and learn a new stroke pattern when the goal is adjusted. Specifically, the reward was reversed at step n = 150, and following the definition set in Figure 2, the robot learned to reverse its F-stroke with adaptation steps of n = 24.6, as shown in the solid blue region. The increased experimentation time allowed the robot to adapt faster to the goal adjustment at n = 150 than it did at n = 0, which had a policy adaptation period of 62.3 steps. B) During the forward reward period, the robot traveled left to right on the substrate. During the reverse reward period, the robot traveled right to left.

Goal adaptation was analyzed by performing experiments similar to the learning robot experiment described earlier, with the addition of a reward modification at step n = 150. To simulate a goal change, the reward function was modified from rewarding positive displacement to negative displacement. During the period of *n* = 0–149, the reward was calculated by Equation (2). The adaptation period was *n* = 62.3 steps. During the period of *n* = 150–300, the reward was calculated by Equation (3). After the change in reward, the robot ceased performing the F-stroke policy within ≈3 steps. The adaptation period was *n* = 24.6 steps. At step n = 161 (11 steps after the reward change), the robot began moving backward, nearly returning to its original position after 150 steps (see Figure 3 and Movie S2, Supporting Information).

### 2.3. Goal Adaptation in Confined Environment

**Figure 4.**
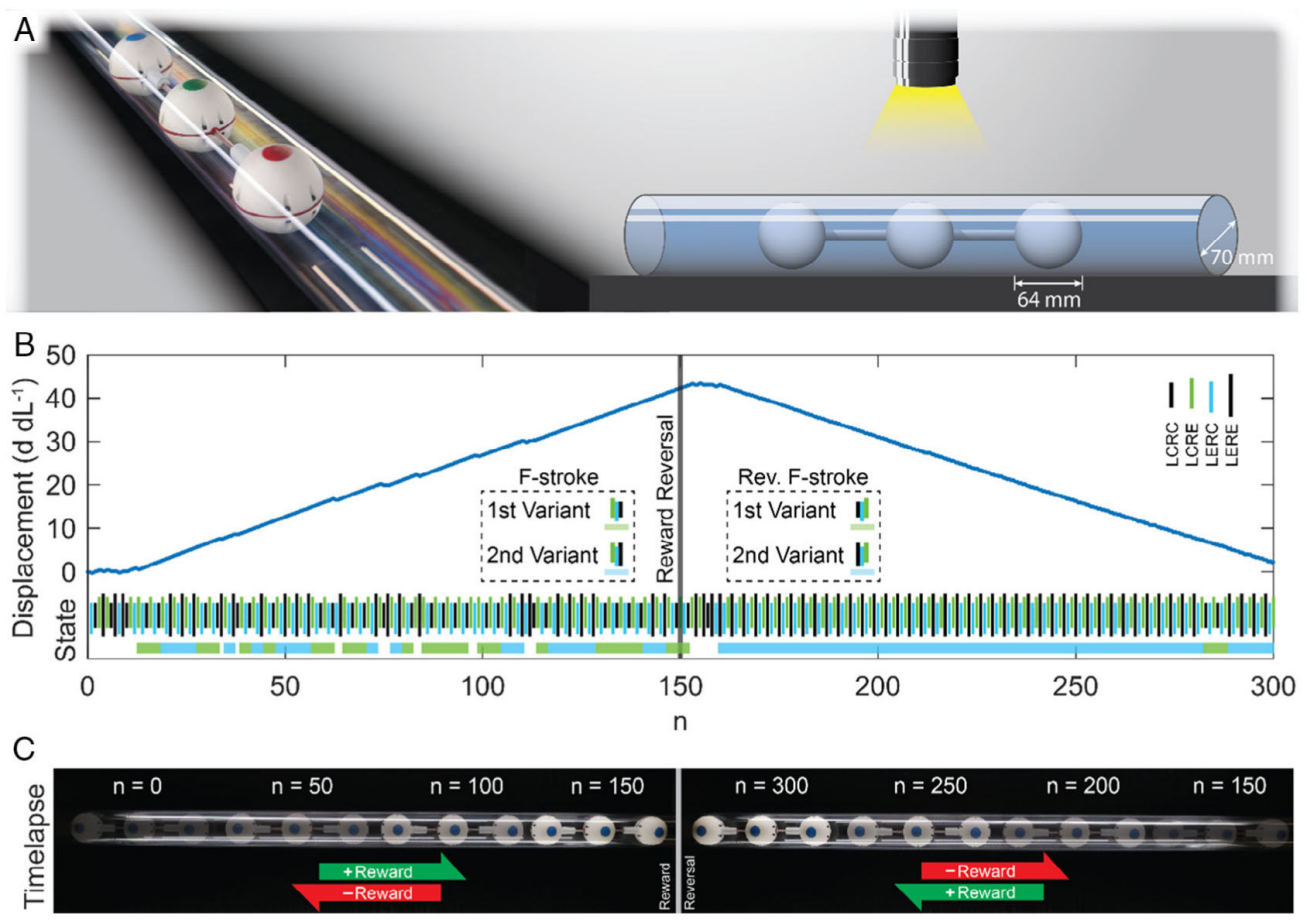
A compact body and robust self-learning behavior allow the robot to navigate through a confined space. A) The robot with an outer diameter of 64 mm was confined to a clear acrylic tube with an inner diameter of 70 mm. The robot’s compact, spherical cross-section and simple push–pull locomotion allow it to navigate through the tube as quickly as the horizontal surface. B) Despite the confinement and the small (≈2%) sliding action, the robot demonstrated the ability to learn to move toward the goal. Similar to the results shown in Figure 3, the robot adapted to the reversal of reward direction with a policy adaptation period of n = 17.4 steps. However, the robot could not achieve our standard for adaptation (n ≥ 48 consecutive steps of an exclusive policy) within the initial 150 steps. C) During the forward reward period (n = 0–149), the robot traveled left to right inside the tube. During the reverse reward period (n = 150–300), the robot traveled right to left.

The robot’s ability to navigate in confined environments was examined by performing an experiment similar to the aforementioned goal-adaptation experiment, with the robot instead being placed inside a clear acrylic tube. The algorithm parameters used in this experiment were identical to the open-surface goal adaptation experiment. During testing, a small amount of slippage, which caused an ≈2% reduction in velocity, was observed. In the first half (*n* = 0–150), the robot did not fully adapt within 150 steps (see Figure 4 and Movie S3, S4, Supplementary Information). After the reward was reversed (at step n = 150), the robot learned the reverse F-stroke in *n* = 17.4 steps.

## 3. Discussion

In this work, several experiments were performed to demonstrate the robot’s ability to 1) adapt movement policy; 2) adapt to goal changes; and 3) adapt to policy and goal changes in a confined environment. In each of the experiments, reinforcement learning allowed the learning robot to learn a productive stroke sequence in ≈54 steps without prior knowledge of the environment. However, even before fully adapting its movement policy, the robot began to make steady forward progress after just 16 steps. Comparing the rates of normalized displacement over many steps, the learning robot achieved ≈90% efficiency compared to a previously optimized crawler while maintaining adaptability.

The ability of reinforcement learning to adapt the robot’s gait from an unproductive N—G stroke to a productive F-stroke can be seen by comparing the displacement curves between the learning and nonlearning robots in Figure 2a. The learning robot identified two variations of the F-stroke, both used by the robot in this and other experiments. The first F-stroke variant consists of the three-state sequence LCRE–LERC–LCRC (L: left link, R: right link, C: contracted, E: extended). The second variant is similar to the first, with an LERE substituted for the third state. Due to symmetry in displacement when performing the two F-stroke variants’ final actions, both the immediate and long-term rewards are identical. Thus, the robot is likely to favor both equally, barring any mechanical irregularities. Over many hours of experiments, the robot’s preference of one variant over the other can vary due to mechanical irregularities causing a slight imbalance in the stroke length (≈±0.48mm) and actuation speed between the two joints (see Figure 2,3,4). The representative experiments shown in the figures are cases where the robot settled on the second variant of the F-stroke.

The relationship between the robot’s state sequence and the center-of-mass displacement is shown in the plot inset to Figure 2a. The inset plot shows that the state progression LERE–LCRC (*n* = 77–78) produced no net displacement, whereas LCRE–LCRC (*n* = 79–80) produced negative displacement, causing the robot to backtrack, and the sequence LCRE–LERC–LCRC (*n* = 81–83) produced a net positive displacement. The small plateaus in displacement located at the end of each step are where the robot briefly rested before the following command.

After demonstrating gait adaptation in response to displacement-driven rewards, the next set of experiments demonstrated the user’s ability to modify robot behavior through goal-driven control. Unlike optimal control, which directly controls the position or effort of a system, reinforcement learning can be used to indirectly guide the robot through positive reinforcement. In the second set of experiments (shown in Figure 3), the robot exhibited bidirectional motion as the 1D reward was modified from awarding positive to negative displacement. As evident in Figure 3a, the displacement and state sequence on either side of the reward reversal line (*n* = 150) show the policy adaptation to the goal adjustment. The robot ceased its left-to-right trajectory and learned to move right to left. During the second half of the goal adaptation experiments (*n* = 150–300), the robot adapted more quickly to a single policy (within ≈17–25 steps), compared to ≈54–62 steps during the first haft (*n* = 0–150), indicating that the speed of the learning behavior is improved when the robot has identified policies, even if those policies are disadvantageous. After *n* = 172, the robot exhibited fewer fluctuations between F-stroke variants, which is most likely due to the increased number of steps allowing the learning algorithm to further reinforce a single policy.

The concept of controlling robot behavior via goal manipulation could be extended beyond the 1D reward change implemented in this work. For example, after updating the robot design, movement in additional dimensions such as 2D planar movement and turning motions could be incentivized with different goals. Similarly, robot configuration and behaviors, such as maintaining a compact body or optimizing efficiency, could also be incentivized. Directing the robot’s motion via goal manipulation is advantageous because it allows the operator to provide real-time guidance without requiring manual supervised control of the robot’s behavior, thus allowing the robot to autonomously adapt its motion toward its goal (e.g., locomotion toward a target) even in a dynamic environment.

## 4. Conclusion

This work presents a 3D-printed three-linked-sphere robot that, using reinforcement learning, was able to identify favorable gait policies and adapt to goal changes within a confined space. Experimental results show the robot could identify favorable stroke sequences for bidirectional locomotion across frictional surfaces in open and confined spaces without previous knowledge of the environment or optimal movement policy. Further, these experiments verify previous theoretical and simulation models of the crawling motion of three-linked-sphere robots and offer potential insights into the applicability of the general design and control strategy in a variety of environments.

Experiments were performed to demonstrate the robot’s ability to learn effective gait policies and adapt to goal adjustments across open surfaces and confined spaces. First, gait policy adaptation in 1D motion was confirmed by comparing a learning robot’s and nonlearning robot’s motion. The experiment simulated a change in the environment by programming the learning robot to use the N—G stroke for the first 20 steps, which is optimized for swimming in low Reynolds numbers and is ineffective for crawling. The reinforcement-learning reward function was then altered to reward positive displacement. Results showed the learning robot adapted its gait policy and identified two F-stroke variations to move forward, whereas the nonlearning robot was unable to adapt or achieve net displacement using the preprogrammed N—G stroke. Reinforcement learning also allowed the robot to maintain flexibility in its movement policies while preserving ≈90% efficiency compared to an optimized, nonlearning frictional crawler.

Next, goal adaptation was demonstrated by reversing the reinforcement-learning reward function to reward backward displacement and observing the robot’s corresponding motion reversal. This experiment demonstrates the ability to control robot motion indirectly using reward function modification, which may be advantageous compared to direct control methods. After reward reversal, it was observed that the robot adapted more quickly to achieve favorable locomotion, which was likely due to the reinforcement-learning algorithm improving the accuracy of the *Q* values over time.

After demonstrating gait adaptation and control via goal manipulation across horizontal surfaces, experiments were performed in a cylinder to simulate a confined environment. As was demonstrated across horizontal surfaces, the learning robot was able to adapt its movement policy and identify the two F-stroke variations in a confined space to achieve net forward displacement and adapt to the goal changes to achieve bidirectional locomotion. Though a small amount of slippage was observed in the confined experiments, the robustness of the reinforcement-learning control enabled the robot to perform similarly in the cylinder as it had on the horizontal surface.

In conclusion, we demonstrate that the synergistic integration of a highly scalable, compact three-linked-sphere robot with model-free reinforcement-learning control can enable adaptable, autonomous navigation in both unconfined and confined environments. The three-linked-sphere robot uses a simple, one-degree-of-freedom actuation that can be miniaturized to a subcentimeter scale. For example, in contrast to COTS, the size of the actuator can be significantly reduced using piezoelectric and shape memory alloy^[44-46]^ and recently developed 3D-printed actuators, which use shape memory polymers, hygroscopic and thermal-responsive composite hydrogels, liquid crystal elastomers, and magnetic composite materials to produce soft robots with tailored deformation.^[47,48]^ The integration with recent work in advanced manufacturing for electronics, such as with 3D printing,^[49-54]^ or conformal electronics^[43,55]^ can be used to reduce the footprint of the electronics packaging (e.g., by distributing the components with freeform, 3D interconnects,^[56-58]^ printed active electronics,^[41,59]^ and printed batteries^[60-62]^).

Another future direction to develop the robot’s ability to navigate confined spaces, including the human body, is to incorporate sensors that can provide feedback to the Q-learning algorithm without requiring a visual connection. The three-linked-sphere robot could be localized through nonvisual means^[63,64]^ such as magnetics^[65,66]^ and radio.^[67]^ We anticipate that untethered robots with these features may be used for long-term monitoring, noninvasive surgery, and targeted therapies in previously inaccessible, confined environments in the human body.

## 5. Experimental Section

### Robot Design:

The three-linked-sphere robot consists of three equally sized spheres that are linearly coupled with two prismatic joints (see Figure 1). The body of each sphere was 3D printed from polyvinyl acrylate (PLA, Ultimaker 3) and thermoplastic polyurethane (TPU, Ultimaker 3), and the joints were 3D printed from photocurable resin (Clear Resin, FormLabs Form 3). The 64 mm spheres contained microcontrollers (ESP WROOM-32) and custom circuit boards for robot control and communication, batteries (1S LiPo, Turnigy Nano-tech), and linear actuators (PQ12, Actuonix). Tungsten powder was added to each sphere to make a heavy ballast for added stability. The top of each sphere was marked with a circle of acrylic paint for vision tracking by an overhead camera. During experiments, the microcontrollers within the robot controlled the prismatic joints and communicated via Bluetooth with an external computer which performed the visual tracking and reinforcement learning.

### Experiment Setup and Terminology:

For unconfined experiments, the robot was placed on a horizontal, level surface, including ultra-high molecular weight polyethylene or aluminum slotted rail. For confined experiments, the robot was placed inside one end of a clear acrylic tube (tube diameter was 109% of robot sphere diameter), which was rigidly fixed to the table surface. Across all experiments, lighting was adjusted to facilitate visual tracking, including through the clear tube’s surface. A left-to-right convention was used in all experiments to define positive displacement, and right to left for negative displacement.

Each robot state was assigned a name based on the joint location and pose as follows: “L” left joint, “R” right joint, “E” extended, “C” contracted. Thus, the state “LCRE” denotes the robot with the left joint contracted and the right joint extended. The robot transitions through the four states (LCRC, LCRE, LERC, LERE; see Figure 2) using 12 state-action pairs. Each state-action pair represents a single stroke. During reinforcement learning, the algorithm learns a sequence of strokes based on a user-defined reward function.

### Vision Tracking and Q-Learning:

During each experiment, images of the robot and surrounding area were captured using a DSLR camera (EOS 80D, Canon) rigidly mounted above the experiment. A circular polarizing filter was used for the confined experiments to reduce the glare from the curved acrylic tube (CIR 67 mm, Tiffen). Images were sent to an external computer, which performed image visual tracking and determined the next step using a Q-learning reinforcement-learning algorithm. Commands to perform the next step were then sent to the robot via Bluetooth.

Visual tracking was performed using a custom MATLAB script that identified tracking markers (colored circles) on each sphere to determine each sphere’s position. These positions were averaged to find the robot’s center of mass, which was used as the position of the robot as a whole. Images were captured at a rate of 24 frames per step, with information from each image being used to calculate robot displacement for analysis. The reinforcement learning algorithm used only the information from the first image per step.

After each step, the Q-learning algorithm was updated and used to calculate the next optimum state-action pair to be performed by the robot. At the beginning of the experiment, the Q-table values were initialized to zero. Then, between each step, updates to the Q-learning algorithm consisted of updating a matrix of 12 values, called a Q-table, whose rows represent the robot’s states and whose columns represent the three actions that transition the robot from the current state to the other states. The algorithm determined the following action by selecting the highest value in the current state (matrix row). In a tie case, the algorithm selected the tied action that appeared left-most in the matrix row.

The Q-table values were updated based on the policy shown in Equation 1, where *s*_*n*_ and *a*_*n*_ are the state and action, respectively, at step *n*. In each experiment, the learning rate, *α*, and discount factor, *γ*, were held constant at 1.0 and 0.9, respectively. The reward *r*_*n*_ was calculated as shown in Equation (2), where *d*_*n*_ and *d*_*n*−1_ are the displacement of the robot’s center of mass, normalized by the joint stroke length, at step *n* and *n* − 1, respectively. Equation (2) was used for the gait adaptation experiments (Figure 2) and during steps *n* = 0–149 of the goal adaptation experiments (Figure 3,4), because it rewards movement in the left–right direction. Equation (3) reverses the displacement reward, rewarding right–left movement. The movement penalty remains the same. As such, Equation (3) was used during steps *n* = 150–300 of the goal adaptation experiments (Figure 3,4) to incentivize the robot to move right to left.


(1) $$Q(s_{n},a_{n})\leftarrow Q(s_{n},a_{n})+\alpha \left[r_{n}+\gamma \max_{a_{n+1}}Q(s_{n+1},a_{n+1})-Q(s_{n},a_{n})\right]$$

(2) $$r_{n}=\frac{\left(d_{n}-d_{n-1}\right)}{20}-0.25$$

(3) $$r_{n}=-\frac{\left(d_{n}-d_{n-1}\right)}{20}-0.25$$

The constants in Equation (2) and (3) were chosen to bring the reward components to similar values. The displacement scalar, 1/20, converts the displacement’s raw pixel value to ≈0.5–0.7, plus or minus a few tenths. The movement penalty, −0.25, penalizes each movement, ensuring that the robot views movement as an inherently costly action, though not to overwhelm the reward from positive displacement. This means that the robot is disincentivized from performing actions that avoid negative displacement but do not achieve positive displacement. Combined with the forward-looking algorithm in Equation (1), the *Q* value stabilized around ≈−0.4.

Robot performance was measured using the number of steps for policy adaptation and average displacement rate. Policy adaptation—when the robot learns a specific movement policy (e.g., F-stroke, N—G stroke)—was defined as the period when the corresponding set of *Q* values remains higher than all other sets for *n* ≥ 48 steps. This allows the robot the opportunity to explore all 12 state-action pairs four times. The first step at the beginning of this period was used to define the number of steps required for policy adaptation. The average displacement rate (*d* d*L*^−1^ *n*^−1^) was calculated from the displacement curve (*d* d*L*^−1^) (see blue line in Figure 2), which was scaled by the stroke length (d*L*).

## Supplementary Material

Supplementary Information

Movie S1

Movie S2

Movie S3

Movie S4

## Data Availability

The data that support the findings of this study are available from the corresponding author upon reasonable request.
