# Supplementary material for: A 3D-Printed Self-Learning Three-Linked-Sphere Robot for Autonomous Confined-Space Navigation
Source: Adv Intell Syst. Author manuscript; Available in PMC 2022 Mar 29. (PMC8963778; doi:10.1002/aisy.202100039)
Supplement: Supplementary Information [file NIHMS1741123-supplement-Supplementary_Information.pdf]

Supporting Information

A 3D printed self-learning three-linked sphere robot for autonomous confined space navigation

*Brian Elder, Zonghao Zou, Samannoy Ghosh, Oliver Silverberg, Taylor Greenwood, Ebru Demir, Vivian Song-En Su, On Shun Pak, Yong Lin Kong**

Movie S1

Video of experiment depicted in Figure 2. Top video corresponds to the learning robot (blue displacement curve, Figure 2). Bottom video corresponds to the non-learning robot (red displacement curve, Figure 2).

Movie S2

Video of experiment depicted in Figure 3. Top video corresponds to the learning robot performing goal adaptation. Bottom video corresponds to the non-learning robot (red displacement curve, Figure 2).

Movie S3

Video of a representative experiment that was similar to the one depicted in Figure 4. Top video corresponds to the confined learning robot performing goal adaptation. Bottom video corresponds to the confined non-learning robot. Using the same experimental parameters, this high-framerate video is meant to complement the low-framerate video of the actual experiment depicted in Figure 4 (see Supplementary Video 1).

Movie S4

Video of the experiment depicted in Figure 4. This video corresponds to the confined learning robot performing gait adaptation. The white crosses mark the sphere positions that were identified by the visual tracking. The mean average of the points is taken as the center-of-mass of the robot.
